# Improved BM212 MmpL3 Inhibitor Analogue Shows Efficacy in Acute Murine Model of Tuberculosis Infection

**DOI:** 10.1371/journal.pone.0056980

**Published:** 2013-02-21

**Authors:** Giovanna Poce, Robert H. Bates, Salvatore Alfonso, Martina Cocozza, Giulio Cesare Porretta, Lluís Ballell, Joaquin Rullas, Fátima Ortega, Alessandro De Logu, Emanuela Agus, Valentina La Rosa, Maria Rosalia Pasca, Edda De Rossi, Baojie Wae, Scott G. Franzblau, Fabrizio Manetti, Maurizio Botta, Mariangela Biava

**Affiliations:** 1 Istituto Pasteur Fondazione Cenci-Bolognetti, Dipartimento di Chimica e Tecnologie del Farmaco, Sapienza Università di Roma, Roma, Italy; 2 Diseases of the Developing World, Tres Cantos Medicines Development Campus, GlaxoSmithKline, Tres Cantos, Madrid, Spain; 3 Dipartimento di Scienze della Vita e dell’Ambiente, Università degli Studi di Cagliari, Cagliari, Italy; 4 Dipartimento di Biologia e Biotecnologie, Università degli Studi di Pavia, Pavia, Italy; 5 Institute for Tuberculosis Research, University of Illinois at Chicago, Chicago, Illinois, United States of America; 6 Dipartimento Farmaco Chimico Tecnologico, Università degli Studi di Siena, Siena, Italy; University of Padova, Medical School, Italy

## Abstract

1,5-Diphenyl pyrroles were previously identified as a class of compounds endowed with high *in vitro* efficacy against *M. tuberculosis*. To improve the physical chemical properties and drug-like parameters of this class of compounds, a medicinal chemistry effort was undertaken. By selecting the optimal substitution patterns for the phenyl rings at N1 and C5 and by replacing the thiomorpholine moiety with a morpholine one, a new series of compounds was produced. The replacement of the sulfur with oxygen gave compounds with lower lipophilicity and improved *in* *vitro* microsomal stability. Moreover, since the parent compound of this family has been shown to target MmpL3, mycobacterial mutants resistant to two compounds have been isolated and characterized by sequencing the *mmpL3* gene; all the mutants showed point mutations in this gene. The best compound identified to date was progressed to dose-response studies in an acute murine TB infection model. The resulting ED_99_ of 49 mg/Kg is within the range of commonly employed tuberculosis drugs, demonstrating the potential of this chemical series. The *in vitro* and *in vivo* target validation evidence presented here adds further weight to MmpL3 as a druggable target of interest for anti-tubercular drug discovery.

## Introduction

*Mycobacterium tuberculosis*, the causative agent of tuberculosis (TB), infects one third of the world’s population and is the second leading cause of mortality worldwide [1]. The World Health Organization (WHO) estimated that there were 8.5–9.2 million cases of TB and 1.2–1.5 million deaths in 2010, including deaths from TB among HIV-positive people. The current 6-month chemotherapy regimen involves treatment with a combination of 4 drugs (isoniazid, rifampicin, pyrazinamide and ethambutol) for 2 months followed by an additional 4 months with isoniazid and rifampicin alone [2]. Although this regimen can achieve 95% cure rates in clinical trials, global cure rates are much lower mainly because of poor patient compliance and poor quality drugs that give rise to drug-resistant strains and infection relapse cases. Almost 4% of all TB cases globally are estimated to be multi-drug resistant [3], with the number of drug resistant cases increasing annually. Thus, new drugs with shorter and simpler regimens and with bactericidal mechanisms that differ from those of current drugs are needed.

**Figure 1:**
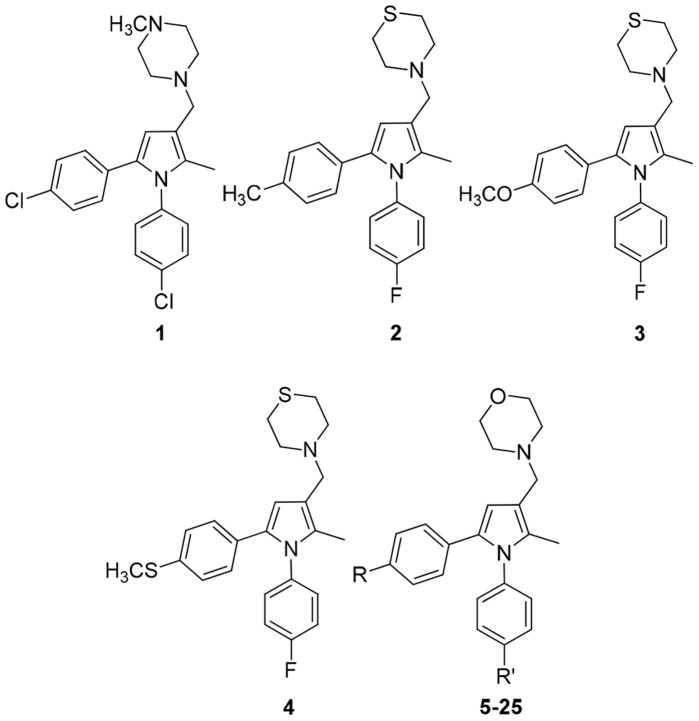
Chemical structures of compounds 1–25.

In this context, a class of 1,5-diphenyl-pyrrole derivatives endowed with potent antimycobacterial activity has been identified through whole-cell screening of a library of azole compounds [4]–[7]. Structure-activity relationship (SAR) studies and a pharmacophore-based ligand design approach allowed us to identify new chemical groups and substitution patterns on the pyrrole core responsible for activity [8]. Our initial SAR strategy evaluated a panel of four compounds **1** (BM212) [7], **2** (BM521) [9], **3** (BM533) [10], and **4** (BM579) [10] (Figure 1) as hits within this class of derivatives on the basis of their biological profiles. Herein, we report our efforts to develop new 1,5-diphenyl pyrrole derivatives with improved drug-like properties as potential new tuberculosis therapeutic agents.

## Materials and Methods

All procedures involving treatment of mice were performed with ethics approval of the Diseases of the Developing World (DDW-GSK) ethical committee. The animal research complies with Spanish and European Union legislation (European directive 86/609/EEC) on animal research and GlaxoSmithKline 3R policy on the care and use of animals: Replacement, Reduction and Refinement.

### Compound Synthesis

Compounds **5–25** were prepared following a previously described synthetic pathway [10]. Please see the Supporting Information for details.

### *Mycobacterium Tuberculosis* H37Rv Growth Inhibition Assay

The measurement of the minimum inhibitory concentration (MIC) for each tested compound was performed in 96 wells flat-bottom, polystyrene microtiter plates. Ten two-fold drug dilutions in neat DMSO starting at 200 µM were performed. Five µL of these drug solutions were added to 95 µL of Middlebrook 7H9 medium. Isoniazid was used as a positive control, 8 two-fold dilutions of Isoniazid starting at 160 µg/mL were prepared and 5 µL of this control curve was added to 95 µL of Middlebrook 7H9 medium (Difco). Five µL of neat DMSO were added 95 µL of Middlebrook 7H9 medium in row 12 (growth and Blank controls). The inoculum was standardized to approximately 1×10^7^ cfu/mL and diluted 1 in 100 in Middlebrook 7H9 broth (Middlebrook ADC enrichment, a dehydrated culture media which supports growth of mycobacterial species available from Becton Dickinson), to produce the final inoculum of H37Rv strain (ATCC25618). One hundred µL of this inoculum was added to the entire plate but G-12 and H-12 wells (Blank controls). All plates were placed in a sealed box to prevent drying out of the peripheral wells and they were incubated at 37°C without shaking for six days. A resazurin solution was prepared by dissolving one tablet of resazurin (Resazurin Tablets for Milk Testing; VWR International Ltd) in 30 mL sterile PBS (phosphate buffered saline). Of this solution, 25 µL were added to each well. Fluorescence was measured (Spectramax M5 Molecular Devices, Excitation 530 nm, Emission 590 nm) after 48 hours to determine the MIC value.

### Low Oxygen Recovery Assay (LORA)

The LORA was performed as previously described [11] except that the low oxygen-adapted inoculum was prepared using a sealed, stirred culture instead of a fermentor. Briefly, low oxygen-adapted *M. tuberculosis* H37Rv *luxAB* was exposed to compounds in 7H12 medium contained in 96-well plates at 37°C under a low oxygen environment generated using an Anoxomat system. After 10 days incubation, plates were placed under normoxic conditions for 28 hours at 37°C and then luminescence was measured following the addition of *n*-decanal. The LORA MIC was defined as the lowest concentration resulting in a 90% reduction in luminescence signal relative to untreated cultures.

### Vero Cytotoxicity Assay

This experiment was carried out as previously described [10]. Briefly, Vero cells were grown and maintained in RPMI 1640 medium supplemented with 2 mM.

L-glutamine and 10% FCS. Cells were seeded in 96-well plates at a density of 1×10^4^ cells/well. After 24 h, medium was replaced with fresh medium containing decreasing concentrations of the tested compound and incubated at 37°C in 5% CO_2_. Morphological changes were observed at 24, 48 and 72 h of incubation. The effects on the proliferation of Vero cells were determined after 72 h by tetrazolium-based colorimetric MTT assay. The 50% cell-inhibitory concentration (CC_50_) reduced by 50% the optical density values (OD_540_,_690_) with respect to control no-drug treated cells.

### HepG2 Cytotoxicity Assay

Actively growing HepG2 cells were removed from a T-175 TC flask using 5 mL of Eagle’s MEM (containing 10% FBS/1% NEAA/1% penicillin + streptomycin) and dispersed in the medium by repeated pipetting. Seeding density should be checked to ensure that new monolayers are not more than 50% confluent at the time of harvesting. Cell suspension was added to 500 mL of the same medium at a final density of 1.2×10^8^ cells per mL. 25 µL of this cell suspension (typically 3000 cells per well) were dispensed into the wells of 384-well clear bottom Greiner plates using a Multidrop. Prior to addition of the cell suspension, these plates were dispensed with 250 nL of the screening compounds using an Echo 555. Plates were allowed to incubate at 37°C and a relative humidity of 80% for 48 hours in the presence of 5% CO_2_. After the incubation period, the plates were allowed to equilibrate at room temperature for 30 minutes before proceeding to develop the luminescent signal. The signal developer, CellTiter- Glo™ (Promega) was equilibrated at room temperature for 30 minutes and added to the plates (25 µL per well) using a Multidrop. The plates were left for 10 minutes at room temperature for stabilization and were subsequently read using a ViewLux (Perkin Elmer).

### Physical Chemical Properties

Standard physical chemical property determination methods were used. Please see the Supporting Information for details.

### Isolation of *M. tuberculosis* H37Rv and *M. bovis* BCG Mutants Resistant to 5 and 8

Mycobacterial strains were grown either in Middlebrook 7H9 broth (Becton Dickinson) supplemented with 0.05% Tween 80 and 10% (vol/vol) oleic acid-albumin-dextrose-catalase (OADC) enrichment (Becton Dickinson), or on 7H11 agar (Becton Dickinson) supplemented with 0.5% glycerol and 10% OADC. Mycobacterial cultures were grown at 37°C without shaking for about three weeks. Compounds **5** and **8** were dissolved in dimethyl sulfoxide. A single colony of mycobacterial strains was inoculated in complete Middlebrook 7H9 and the cultures were incubated at 37°C until reached exponential growth phase. Cultures were diluted to the final concentration of about 10^7^ CFU/mL and 1 µL of dilutions was then streaked onto plates containing twofold scalar dilutions of compounds **5** and **8**. The MIC was defined as the lowest concentration of drug preventing bacterial growth. All experiments were repeated three times.

*M. tuberculosis* and *M. bovis* mutants resistant to compounds **5** and **8** were isolated by plating about 10^10^ cells from late exponential wild-type cultures onto solid media containing different concentrations of each compound, ranging from 5 to 10-fold MIC for the wild-type strain. Plates were incubated at 37°C for 4 weeks. The MIC of compounds **1**, **5**, and **8** for the isolated resistant mutants was evaluated three times.

### Characterisation of *M. tuberculosis* H37Rv and *M. bovis* BCG Mutants Resistant to 5 and 8

*mmpL3* gene of each mutant was amplified by PCR and sequenced. All primers used are listed in Table S1 reported in the supporting information. Amplification reactions were performed in a final volume of 40 µL, containing 200 µM of each dNTP, 500 nM of each primer, 2% DMSO, 2.5 mM MgCl_2_, 100 ng of genomic mycobacterial DNA and 1 U of *Pfu* DNA Polymerase (Promega). Cycling conditions were as follows: denaturation at 95°C for 2 min, followed by 30 cycles of denaturation at 95°C for 1 min, annealing for 30 sec at a temperature dependent on primers used, and elongation at 72°C for a time dependent on products size, with a final elongation at 72°C for 5 min.

### Pharmacokinetic Studies

Species: C57BL/6 mouse, female, 18–20 g; route: po, oral gavage; feeding regimen: fed.; compartment analyzed: peripheral total Blood.

*Rifampicin*. dose level: po dose, 10.4 mg Kg^−1^, 20 ml Kg^−1^ vol. adm.; vehicle po PK (solution): Water-20% Encapsine™; sampling scheme: 5, 15, 30 and 45 minutes, 1, 1.5, 2, 3, 4 and 8 hours; n = 3 mice per time point (population PK).

*Isoniazid*. dose level: po dose, 0.98 mg Kg^−1^, 20 ml Kg^−1^ vol. adm.; vehicle po PK (solution): Milli-Q Water; sampling scheme: 5, 15, 30 and 45 minutes, 1, 1.5, 2, 3, 4 and 8 hours; n = 3 mice per time point (population PK).

*Moxifloxacin.* dose level: po dose, 27.5 mg Kg^−1^, 20 ml Kg^−1^ vol. adm.; vehicle po PK (solution): Water-20% Captisol™; sampling scheme: 10, 20, 30 and 45 minutes, 1, 1.5, 2, 3, 4 and 8 hours; n = 3 mice per time point (population PK).

*Compound* ***9***. dose level: po dose, 50 mg Kg^−1^, 20 ml Kg^−1^ vol. adm.; vehicle po PK (suspension): Water-1% methylcellulose (1% MC); sampling scheme: 15, 30 and 45 minutes, 1, 2, 4, 8 and 24 hours; n = 3 mice (individual PK).

Analytical method, sensitivity: LC/MS, LLQ = 1–5 ng mL^−1^ in 25 µL blood; data analysis: Non-compartmental analysis (NCA) with WinNonlin Phoenix 6.3 (Pharsight, Certara L.P); supplementary analysis with GraphPad Prism 5 (GraphPad Software, Inc).

### Dose-response Studies in an Acute Murine Infection TB Model

This experiment was carried out as previously described [12]. In brief, nine (one per dose) 8–10 week old B6 female mice (Harlan, Barcelona, Spain) were infected by intratracheal route with 10^5^ CFU H37Rv per mouse suspended in 50 µL phosphate buffer saline. Compound **9** was administered once a day at nine doses ranging from 40 to 300 mg/kg from day 1 to day 8 after infection, and 24 hours after the last dose the mice were sacrificed. To measure the infection burden in lungs, all lobes were aseptically removed and homogenized. The homogenates were supplemented with 5% glycerol and stored frozen (−80°C) until plating. Plates (10%OADC-7H11 medium) were incubated for 14 days at 37°C.

## Results and Discussion

As part of our continuous medicinal chemistry efforts to identify more potent and safe anti-mycobacterial agents, four hit structures (**1, 2, 3** and **4,** Figure 1) among the previously synthesized derivatives were evaluated for drug-like properties such as lipophilicity (Chromatographic Hydrophobicity Index LogD,CHILogD), protein binding (Human Serum Albumin, HSA and Plasma Protein Binding, PPB) and clearance in order to improve their chances of success in development. Although compounds **2**, **3** and **4** showed good activities against *M. tuberculosis* (ranging from 0.16 to 0.2 µM, Table 1), they also displayed a significantly higher rate of clearance in mouse microsomal fractions than **1** (Table 2). After identifying the thiomorpholine of compounds **2–4** as a potential cause of microsomal instability, we focused our efforts on modifying this group to improve the clearance data.

**Table 1:** *In vitro* antimycobacterial activity against *M. tuberculosis* H37Rv, activity against *M. tuberculosis* H37Rv in the LORA, cytotoxicity in Vero cells and HepG2 cells, of compounds **1–4**.

**Comp.**	**MIC (µM)** a	**MIC (µM)** b	**CC_50_ (µM)** c	**Tox_50_ (µM)** d
**1**	5	18.5	–	7.8
**2**	0.16	17.80	178.80	24.01
**3**	0.20	10.08	>302.26	>2423.77
**4**	0.16	13.81	15.20	5.90

a antimycobacterial activity against *M. tuberculosis* H37Rv.

b antimycobacterial activity against *M. tuberculosis* H37Rv in the LORA.

c cytotoxicity in Vero cells.

d cytotoxicity in HepG2 cells.

**Table 2:** CHILogD, % HSA binding, % PPB, clearance in mouse microsomes of compounds **1–4**.

**Comp.**	**CHILogD pH 2**	**CHILogD pH 7.4**	**CHILogD pH 10.5**	**HSA (%)**	**PPB (%)** a	**Cli mouse (mL/min/g)**	**T_1/2_ (min)**
**1**	0.7	4.51	5.01	98.07	99.8±0.1	0.7±0.1	>30
**2**	1.31	4.54	5.08	97.91	>99.9	7.2±0.2	8.2
**3**	1.07	3.97	4.62	96.82	99.6	18.76	<5
**4**	1.33	4.52	5.03	98.04	99.3±0.1	7.3±0.1	10

a at 1 (µM).

First, we synthesized compounds **5, 6** and **7** as oxygen containing derivatives of **2, 3** and **4** respectively (Figure 1 and Table 3). To determine the *in vitro* activity under aerobic conditions of the newly synthesized derivatives, *M. tuberculosis* H37Rv was incubated in the presence of a range of different compound concentrations. Additionally, compounds **5–7** were tested against mycobacteria in the physiological state of non-replicating persistence (NRP), which is commonly accepted as being responsible for antimicrobial tolerance in many bacterial infections [13], using a low-oxygen-recovery assay (LORA). The subpopulation of *M. tuberculosis* isolates in NRP can contribute to the length of current treatment; thus, to shorten the currently recommended 6-months regimen, research efforts on this phenotype are important. The cytotoxicity of the compounds was determined using both Human hepatocarcinoma (HepG2) and normal African green monkey kidney epithelial (Vero) cells.

**Table 3:** Chemical structure, *in vitro* antimycobacterial activity against *M. tuberculosis H37Rv*, activity against *M. tuberculosis* H37Rv in the LORA, and cytotoxicity in Vero cells and HepG2 cells of compounds **5–25**.

**Comp.**	**R**	**R^’^**	**MIC (µM)** a	**MIC (µM)** b	**CC_50_ (µM)** c	**Tox_50_ (µM)** d	**Tox_50_/MIC**
**5**	-CH_3_	F	0.3	55.56	>351.20	20.08	66.93
**6**	-OCH_3_	F	0.6	>13.4	58.68	>25	>41.66
**7**	-SCH_3_	F	0.6	72.47	44.34	32.9	54.83
**8**	-C_2_H_5_	F	0.2	50.04	18.89	19.5	97.5
**9**	- *^i^*C_3_H_7_	F	0.12	28.73	50.15	15.3	127.5
**10**	-CH_3_	Cl	0.6	42.45	179.26	25	41.66
**11**	-C_2_H_5_	Cl	0.2	>320.52	>256	8.8	44
**12**	-C_3_H_7_	Cl	2	31.66	>256	13.3	6.65
**13**	- *^i^*C_3_H_7_	Cl	0.6	24.74	240.88	10.0	16.66
**14**	Cl	-CH_3_	1.3	30.76	9.307	26.0	20
**15**	Cl	-C_2_H_5_	0.6	16.83	170.33	17.8	29.66
**16**	Cl	-C_3_H_7_	0.6	29.70	60.14	15.4	25.66
**17**	Cl	- *^i^*C_3_H_7_	0.9	6.74	175.44	14.7	16.33
**18**	-CH_3_	-OCH_3_	2	140.84	207.79	11.7	5.85
**19**	-C_2_H_5_	-OCH_3_	0.3	54.66	30.01	5.7	19
**20**	-C_3_H_7_	-OCH_3_	0.3	28.82	>256	14.3	47.66
**21**	- *^i^*C_3_H_7_	-OCH_3_	0.3	23.82	28.23	14.1	47
**22**	-OCH_3_	-CH_3_	0.6	65.86	50.15	11.3	18.83
**23**	-OCH_3_	-C_2_H_5_	0.3	40.63	16.03	14.7	49
**24**	-OCH_3_	-C_3_H_7_	0.16	7.31	31.23	2.1	13.12
**25**	-OCH_3_	- *^i^*C_3_H_7_	0.2	17.10	12.94	15.9	79.5
**Isoniazid**	–	–	1.8	>128	>256	–	–
**Rifampin**	–	–	0.02	–	207.03	–	–

a antimycobacterial activity against *M. tuberculosis* H37Rv.

b antimycobacterial activity against *M. tuberculosis* H37Rv in the LORA.

c cytotoxicity in Vero cells.

d cytotoxicity in HepG2 cells.

Although compounds **5, 6** and **7** gave MICs 1–2 dilutions higher than the parent compounds (0.3 µM for **5** *versus* 0.16 µM for **2**, 0.6 µM for **6** *versus* 0.2 µM for **3**, 0.6 µM for **7** *versus* 0.16 µM for **4**, see Tables 1 and 3), these values were counterbalanced by moderate reductions in the HepG2 cytotoxicity data (20.08 µM for **5** *versus* 24.01 µM for **2**, >25 µM for **6** *versus* 15.2 µM for **3**, 32.9 µM for **7** *versus* 5.9 µM for **4**, see Tables 1 and 3). For all the tested compounds, the MICs in the LORA were higher than those obtained under aerobic conditions (>13.4–72.47 µM *versus* 0.3–0.6 µM, see Table 3), a common result for many known anti-TB drugs including isoniazid [11].

Importantly however, the new morphoiline derivatives gave a notable improvement in mouse microsomal stability (Table 4). While not optimal, the value of 3.4 mL/min/g for compound **5** was highly encouraging as it demonstrated the ability to achieve compounds with good potencies that could also realistically be tested in murine models of TB infection [12].

**Table 4:** Clearance in mouse and human microsomes of compounds **5–25**.

**Comp.**	**Cli mouse (mL/min/g)**	**t_1/2_ (min)**	**Cli human (mL/min/g)**	**t_1/2_ (min)**
**5**	3.4±0.2	20.4	<0.5	>30
**6**	4.7±0.1	15.9	0.5±0	>30
**7**	7.30	9.27	0.56	>30
**8**	5.64	12.51	1.09	>30
**9**	1.4±0	>30	<0.5	>30
**10**	2.6±0.1	25.7	<0.5	>30
**11**	2.83	25.04	0.52	>30
**12**	1.49	>30	<0.5	>30
**13**	2.13	>30	0.71	>30
**14**	7.41	9.69	0.57	>30
**15**	4.0±0.1	18.5	0.9±0	>30
**16**	1.2±0	>30	<0.5	>30
**17**	3.8±0.1	19.6	0.5±0	>30
**18**	8.92	<5	1.26	59.85
**19**	9.45	<5	1.71	>30
**20**	5.75	10.86	0.61	>30
**21**	6.07	10.67	2.15	>30
**22**	12.62	<5	1.14	>30
**23**	7.8±0.5	<5	<0.5	>30
**24**	2.1±0.1	>30	<0.5	>30
**25**	10.55	<5	0.5	>30

With these initial encouraging results in hand, a series of morpholine derivates was prepared with different substitution patterns on the N1 and C5 aryl groups (compounds **8–25**). Nearly all the newly synthesized compounds showed good activities ranging from 0.12 to 0.6 µM (Table 3) under aerobic conditions. As before, they proved to be less active under LORA conditions (Table 3). Only compounds **13**, **15**, **17**, **21** and **24** proved to be moderately active against *M. tuberculosis* under LORA conditions with MICs ranging from 6.74 to 24.74 µM (Table 3).

As before, the compounds were then tested for cytotoxicity against Vero and HepG2 cell lines. Compounds **10–13**, **15**, **17**, **18** and **20** showed low levels of activity against Vero cells (ranging from 170.33 to 351.2 µM) but gave more significant HepG2 toxicity (Table 3).

While cytotoxicity, particularly against HepG2, is a cause of concern with this family, a number of analogues (e.g., **9**, **20**, **21**, **23** and **25**) showed sufficient therapeutic windows to continue as prospective drug leads (Tox_50_:MIC ratio of >100, 47.66, 47, 49 and 79.5, respectively, see Table 3).

Evaluation of the drug-like parameters, such as lipophilicity, HSA binding, and PPB showed generally high values across the series, with HSA binding in particular showing a clear correlation with the CHILogDs (Table 5). Compounds **8–11**, **13**, **14** and **18–25** showed the lowest level of HSA binding (ranging from 95.58 to 97.74) while only compounds **8**, **10**, **18**, **19**, and **21–23** gave <99% PPB (Table 5).

**Table 5:** CHILogD, % HSA binding and % PPB of compounds **5–25**.

**Comp.**	**CHILogD pH 2**	**CHILogD pH 7.4**	**CHILogD pH 10.5**	**HSA (%)**	**PPB (%)** a
**5**	1.12	3.86	4.26	96.72	99.5±0.1
**6**	0.93	3.32	3.73	95.58	98.3±0.3
**7**	1.13	3.82	4.24	97.00	99.6
**8**	1.26	4.21	4.64	97.14	99.4
**9**	1.47	4.57	4.95	97.71	>99.9
**10**	1.30	4.40	4.78	97.69	99.8±0.1
**11**	1.42	4.77	5.16	97.74	>99.9
**12**	1.61	5.30	5.59	98.00	>99.9
**13**	1.56	5.13	5.45	97.94	>99.9
**14**	1.28	4.53	4.94	97.62	>99.9
**15**	1.54	5.01	5.36	98.03	>99.9
**16**	1.72	5.61	5.86	98.24	>99.9
**17**	1.67	5.43	5.70	98.19	>99.9
**18**	1.04	3.60	4.12	95.87	99.3
**19**	1.21	3.99	4.50	96.65	99.7
**20**	1.40	4.46	4.93	97.17	>99.9
**21**	1.35	4.32	4.81	97.07	99.9
**22**	1.02	3.57	4.11	95.64	99.2
**23**	1.24	4.05	4.52	96.79	99.8±0.1
**24**	1.46	4.52	4.97	97.43	>99.9
**25**	1.36	4.36	4.86	97.02	>99.9

a at 1 (µM).

An important note with regards to the overall high lipophilicity of this compound series is the significant drop in CHILogD from neutral to acidic pH (Table 5). This change demonstrates the high basicity of the nitrogen of the saturated heterocycle ring and greatly improves the chances of finding suitable formulations for this family. Moreover, along with their high CHILogD values, these compounds are relatively planar, with three aromatic rings. Given the well-established connection between late stage development problems and lipophilicity/aromaticity, future medicinal chemistry efforts will be focused on reducing logD values and the number of aromatic rings. A CHILogD_7.4_ value <3 will be targeted, along with the removal of one or more aromatic rings.

As we had hoped from compounds **5–7**, the morpholine series provided consistently improved microsomal clearance values relative to the thiomorpholines, with some derivatives (e.g., **9**, **12**, **13**, **16** and **24**) achieving very promising levels of ≤2 mL/min/g and desirable half lives of >30 min (Table 4). It is also worth noting that the human microsomal stabilities were consistently good.

**Figure 2:**
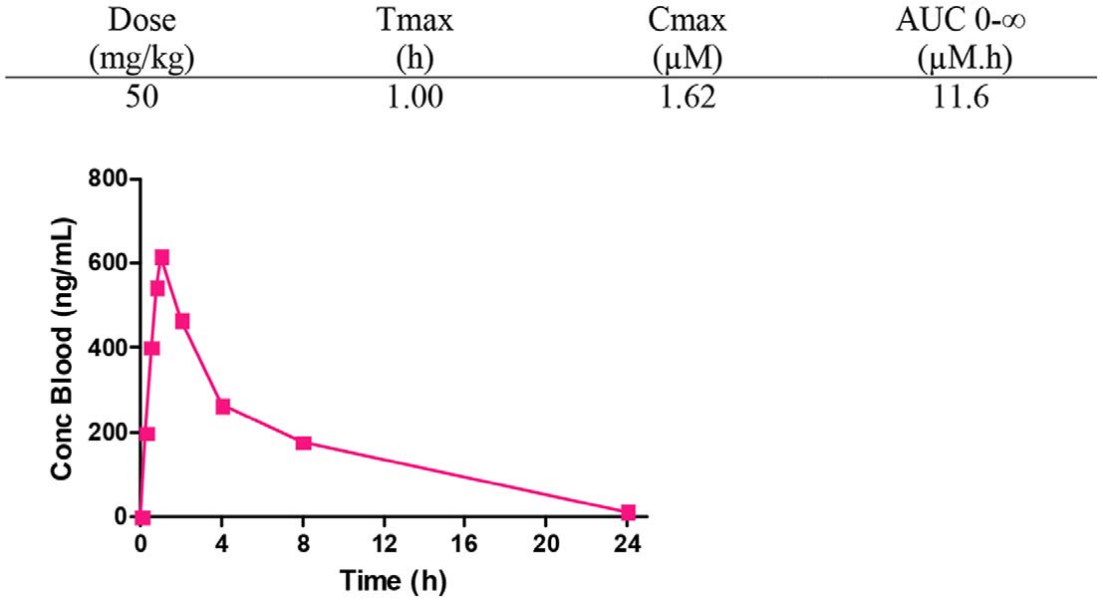
Peripheral blood levels of compound 9 after oral administration to C57BL/6 mice (n = 3) at 50 mg/kg, as a suspension in 1% methyl cellulose; Tlast = 24 h.

**Figure 3:**
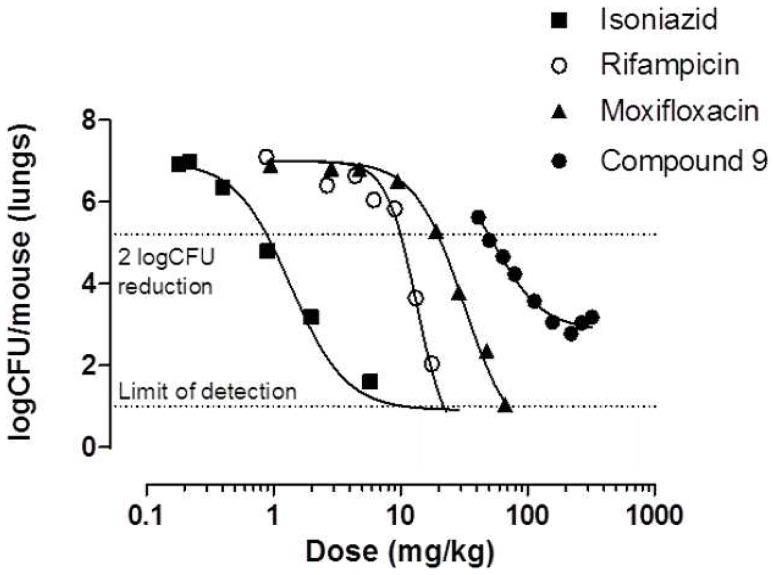
Acute infection model dose-response curve correlating logCFU count reduction in the lungs of mice with different doses of rifampicin, isoniazid, moxifloxacin, and compound 9.

To better characterize the potential value of this chemical series, compound **9** was selected for *in vivo* pharmacokinetic (PK) and efficacy studies (Figure 2). Compound **9** was chosen based on its potent MIC (0.12 µM), Tox_50_:MIC ratio of >100, and good microsomal stability in mice (1.4 mL/min/g). Gratifyingly, when tested in an acute murine infection model [12] at multiple doses, compound **9** exhibited potent anti-tubercular activity *in vivo*, with an ED_99_ (Efficacious Dose that results in a 99% CFU reduction in the lung) of 49 mg/Kg (IC_95%_: 43–54 mg/Kg) (Figure 3). While this ED_99_ is somewhat higher than those of current standards of care (Table 6), it represents a strong starting point for a new anti-tubercular lead compound.

**Table 6:** A direct comparison in terms of the Areas Under the Curve (AUC) and oral doses associated with a 99% reduction of cfu counts in the lungs of infected mice (ED_99_) compared to untreated control for rifampicin, isoniazid, moxifloxacin and compound **9**.

**Compound**	**Vehicle**	**ED_99_ (mg/kg)** a	AUC_inf_ (µM*h)
**Isoniazid**	Water	0.95 (0.86–1.1 )	5.5^b^
**Rifampicin**	Water 20%Encapsine™	9.8 (9.0–11)	102.0^c^
**Moxifloxacin**	Water-20%Captisol™	28 (26–29)	9.4^d^
**9**	Water–1%methylcellulose	49 (43–54)	11.6^e^

a Dose that reduces 2 logs bacterial burden in the lungs of mice (acute phase). Data are calculated from individual log_10_CFU/lungs fitted to a logistic equation. Data are expressed as ED_99_ (mg/kg) and the confidence interval of ED_99_ at 95% (in parenthesis).

b AUCinf of 0.98 mg/Kg single oral dose.

c AUCinf of 10.4 mg/Kg single oral dose.

d AUCinf of 27.5 mg/Kg single oral dose.

e AUCinf of 50 mg/Kg single oral dose.

To better understand the true value of this *in vivo* efficacy result, the oral pharmacokinetic profile of compound **9** was determined at 50 mg/kg (Figure 2). The half life *in vivo* of the compound was 1 h, allowing a reasonable maximum concentration (C_max_ = 1.62 µM) and overall exposure. Importantly, when the areas under the curve (AUC_inf_) associated with their respective ED_99_ values were compared, the potency per effective concentration *in vivo* of compound **9,** was shown to be competitive with the standard anti-tuberculars rifampicin, isoniazid, and moxifloxacin (Table 6). By this measure, isoniazid provides the lowest blood exposure needed to reduce the bacterial load by two log units (5.5 µM.h), but compound **9** achieves this important value at a very similar concentration to moxifloxacin (11.6 versus 9.4 µM.h, respectively). This evidence leads us to believe that further optimization of some key medicinal chemistry parameters (e.g., lipophilicity and aromaticity) should provide new leads with enhanced drug-like properties, thus leading to improved potency *in vivo* at lower doses. Efforts along these lines are underway and will be reported in due course.

The isolation and characterization of *M. smegmatis* mc^2^155, *M. bovis* BCG and *M. tuberculosis* H37Rv mutants resistant to compound **1** allowed us to identify MmpL3, as the possible cellular target of these 1,5-diphenyl pyrroles [14]. The *mmpL3* gene was first suggested to be essential for the growth of *M. tuberculosis* [15] and its essentiality was confirmed for *M. smegmatis* too [16], [17]. Furthermore, it has been shown that MmpL3 is a transporter of trehalose mono-mycolate in *M. tuberculosis*, the precursor of trehalose dimycolate and cell wall mycolates [16], [17].

Compounds **5** and **8** were chosen as examples for this new series of derivatives for isolation and characterization of *M. tuberculosis* H37Rv and *M. bovis* BCG mutant resitors. Plating of mycobacterial cultures onto solid media containing varying concentrations of compounds **5** and **8** yielded multiple isolates, with a frequency of approximately 3 in 10^7^ cells. *M. tuberculosis* isolates resistant to compound **5** (DR4-DR9) showed MIC values ranging from 0.68 to 5.4 µM –4 to 33 folds higher than the parent strain (Table 7). *M. tuberculosis* isolates resistant to compound **8** (DR1-DR3) showed an MIC value of 0.66 µM – about 17 fold higher than the parent strain (Table 7). No *M. bovis* BCG mutants resistant to compound **5** were isolated, while two isolated mutants (M1 and M2) resistant to compound **8** showed MICs of 0.63 µM –16 fold higher than the parent strain (Table 7). DR4-DR9 mutants resistant to compound **5**, DR1-DR3 and M1 and M8 mutants resistant to compound **8** were then tested for cross resistance to **8** and **5**, respectively, as well as to compound **1**.

**Table 7:** MIC (µM) of compounds **1**, **5**, and **8** and amino acid substitutions in the *mmpL3* genes of *M. tuberculosis* H37Rv and *M. bovis* BCG mutants isolated as resistant to compound **5** (DR4-DR9) and **8** (DR1-DR3, M1 and M8).

*M. tuberculosis* strains	MIC (µM)			Amino acid change
	1	5	8	
**H37Rv wild-type**	3.6	0.16	0.04	–
**DR1**	14.5	5.4	0.66	V681I
**DR2**	14.5	5.4	0.66	V681I
**DR3**	14.5	5.4	0.66	V681I S87P
**DR4**	14.5	5.4	0.66	V681I V581A N365S
**DR5**	14.5	2.7	0.66	G253E D46G
**DR6**	14.5	5.4	0.66	V681I M492T V564A
**DR7**	14.5	0.68	0.16	Q40R
**DR8**	14.5	2.7	0.33	G253E I516T
**DR9**	14.5	1.35	0.33	V240M R735C
***M. bovis*** ** BCG strains**				
***M. bovis*** ** BCG wild-type**	1.88	0.08	0.04	–
**M1**	15.04	2.6	0.63	L320R E466K I585V
**M8**	15.0	2.6	0.63	L320P S258G

To characterize these mutants, the *mmpL3* gene was amplified and sequenced, revealing point mutations which caused amino acid changes (Table 7). It is worth noting the relationship between the level of drug resistance and the presence of an amino acid substitution. Among the *M. tuberculosis* mutants, the isolates showing higher MICs of **5** (5.4 µM) confirmed the same amino acid change (V681I), even if mutants DR3, DR4, and DR6 presented other amino acid changes (Table 7).

*M. tuberculosis* mutants DR5 and DR8 showed the amino acid change G253E, which was also identified in resistance to an adamantyl urea compound (AU1235) targeting MmpL3 protein [17]. Similarly, the amino acid change Q40R, found in *M. tuberculosis* mutant DR7, seems to be involved in resistance to compound SQ109, a tuberculosis drug candidate targeting MmpL3 [18]. Compounds **8** and **5** showed similar MICs for DR5 and DR8, while MICs of both **5** and **8** for mutant DR7 were lower with respect to the general trend (Table 7), suggesting that the amino acid change Q40R is less important in the interaction with the two compounds.

Additionally, the *mmpL3* genes from *M. bovis* mutants M1 and M8 resistant to compound **8** were also amplified and sequenced. Together with other amino acid substitutions, these two mutants showed a mutation in *mmpL3* resulting in a change from leucine to proline or arginine at position 320 (L320P or L320R) (Table 7). Of note, this amino acid change occurred in 14 out of 15 *M. bovis* BCG mutants resistant to **1** [14], suggesting that this residue is important for conferring resistance to both **1** and **8**. Furthermore, this amino acid change also characterized a *M. tuberculosis* mutant resistant to the antitubercular compound C215, also targeting MmpL3 protein [18].

Surprisingly, among the compounds targeting MmpL3, only these 1,5-diphenyl pyrroles showed some activity against non-replicating mycobacteria (Tables 1 and 3). In fact, neither AU1235 nor SQ109 gave detectable activity against *M. tuberculosis* H37Rv bacilli in anaerobic models [17]–[19]. Moreover, Zhang et al. pointed out that there is a cell wall inhibitor signature; by screening different compounds affecting cell wall biosynthesis against non-replicating bacilli they proved that none of the compounds showed detectable activity against non-growing bacteria [20]. These results may suggest that these 1,5-diphenyl pyrroles also target a biosynthetic pathway required for non-growing bacteria, though further studies are needed.

### Conclusions

We have demonstrated that the bio-isosteric replacement of the sulfur atom with oxygen retains the activity of 1,5-diphenyl pyrroles against *M. tuberculosis* and provides general improvements in the physicochemical properties of these compounds. Spontaneous resistant mutant characterization with compounds **5** and **8** confirmed MmpL3 as the potential target of the pyrrole anti-tuberculars. This inhibitor class has also recently been expanded to another three compound families [17]–[19]. The encouraging *in vivo* efficacious response found in a murine model of TB infection provides further evidence of the attractiveness of the pyrroles and potentially of other MmpL3 inhibitors for additional lead optimization activities.

## Supporting Information

Figure S1 **Synthetic scheme for compounds 5–25.**
(TIF)

Table S1 **Oligonucleotides used in this work.**
(PDF)

Protocol S1 **Procedure for preparation of compounds 5–25. Physicochemical data of compounds 5–25.**
(PDF)

Protocol S2 **HPLC analysis of compounds 5–25.**
(PDF)

Protocol S3 **Determination of compounds Chromatographic Hydrophobicity Index (CHI) at acidic, neutral and alkaline pHs.**
(PDF)

Protocol S4 **Determination of compounds binding to human serum albumin (HSA).**
(PDF)

Protocol S5 **Determination of compounds binding to plasma proteins (PPB).**
(PDF)

Protocol S6 **Microsomal fraction stability experimental procedure.**
(PDF)

## References

[1] Global Tuberculosis Control: WHO report 2011, World Health Organization, Geneva (WHO/HTM/TB/2011.16).

[2] Treatment of Tuberculosis: guidelines for national programmes 4^th^ ed., World Health Organization, Geneva (WHO/HTM/TB/2009.420).

[3] Multidrug and extensively drug-resistant TB (M/XDR-TB): 2010 global report on surveillance and response (2010) World Health Organization, Geneva (WHO/HTM/TB/2010.3).

[4] Biava M, Fioravanti R, Porretta GC, Sleiter G, Ettorre A, et al (1997) New toluidine derivatives with antimycobacterial and antifungal activities. Med Chem Res 7: 228–250.

[5] Fioravanti R, Biava M, Donnarumma S, Porretta GC, Simonetti M, et al (1996) Synthesis and microbiological evaluation of (*N*-heteroaryl)arylmethanamines and their Shiff bases. II Farmaco 51: 643–652. 8981754

[6] Fioravanti R, Biava M, Porretta GC, Artico M, Lampis G, et al (1997) *N*-substituted 1-aryl-2(1H-imidazol-1-yl)1-ethanamines with broad spectrum *in vitro* antimycobacterial and antifungal activities. Med Chem Res 7: 87–97.

[7] Deidda D, Lampis G, Fioravanti R, Biava M, Porretta GC, et al (1998) Bactericidal activities of the pyrrole derivative BM 212 against multidrug-resistant and intramacrophagic *Mycobacterium tuberculosis* strains. Antimicrob Agents Chemother 42: 3035–3037. 9797251 10.1128/aac.42.11.3035 PMC105991

[8] Biava M, Porretta GC, Poce G, Battilocchio C, Alfonso S, et al (2011) Developing pyrrole-derived antimycobacterial agents: a rational lead optimization approach. Chem Med Chem 6: 593–599. 21341373 10.1002/cmdc.201000526

[9] Biava M, Porretta GC, Poce G, Supino S, Deidda D, et al (2006) Novel diarylpirrole derivatives of BM212 endowed with high activity toward *Mycobacterium tuberculosis* and low cytotoxicity. J Med Chem 49: 4946–4952. 16884306 10.1021/jm0602662

[10] Biava M, Porretta GC, Poce G, Battilocchio C, Alfonso S, et al (2010) Identification of a novel pyrrole derivative endowed with antimycobacterial activity and protection index comparable to that of the current antitubercular drugs streptomycin and rifampin. Bioorg Med Chem 18: 8076–8084. 20934344 10.1016/j.bmc.2010.09.006

[11] Cho SH, Warit S, Wan B, Hwang HH, Pauli GF, et al (2007) Low-oxygen-recovery assay for high-throughput screening of compounds against nonreplicating *Mycobacterium tuberculosis.* . Antimicrob Agents Chemother 51: 1380–1385. 17210775 10.1128/AAC.00055-06 PMC1855511

[12] Rullas J, García JI, Beltrán M, Cardona P-J, Cáceres N, et al (2010) Fast standardized therapeutic-efficacy assay for drug discovery against tuberculosis. Antimicrob Agents Chemother 54: 2262–2264. 20160054 10.1128/AAC.01423-09 PMC2863680

[13] Coates A, Hu Y, Bax R, Page C (2002) The future challenges facing the development of new antimicrobial drugs. Nat Rev Drug Discov 1: 895–910. 12415249 10.1038/nrd940

[14] La Rosa V, Poce G, Ortiz Canseco J, Buroni S, Pasca MR, et al (2012) MmpL3 is the cellular target of the antitubercular pyrrole derivative BM212. Antimicrob Agents Chemother 56: 324–331. 22024828 10.1128/AAC.05270-11 PMC3256021

[15] Domenech P, Reed MB, Barry CE 3rd (2005) Contribution of the *Mycobacterium tuberculosis* MmpL protein family to virulence and drug resistance. Infect Immun 73: 3492–3501. 15908378 10.1128/IAI.73.6.3492-3501.2005 PMC1111821

[16] Varela C, Rittmann D, Singh A, Krumbach K, Bhatt L, et al (2012) MmpL genes are associated with mycolic acid metabolism in Mycobacteria and Corynebacteria. Chem Biol 19: 498–506. 22520756 10.1016/j.chembiol.2012.03.006 PMC3370651

[17] Grzegorzewicz AE, Pham H, Gundi VA, Scherman MS, North EJ, et al (2012) Inhibition of mycolic acid transport across the *Mycobacterium tuberculosis* plasma membrane. Nat Chem Biol 8: 334–341. 22344175 10.1038/nchembio.794 PMC3307863

[18] Tahlan K, Wilson R, Kastrinsky DB, Arora K, Nair V, et al (2012) SQ109 targets MmpL3, a membrane transporter of trehalose monomycolate involved in mycolic acid donation to the cell wall core of *Mycobacterium tuberculosis* . Antimicrob Agents Chemother 56: 1797–1809. 22252828 10.1128/AAC.05708-11 PMC3318387

[19] Stanley SA, Grant SS, Kawate T, Iwase N, Shimizu M, et al (2012) Identification of novel inhibitors of *M. tuberculosis* growth using whole cell based high-throughput screening. ACS Chem Biol 7: 1377–1384. 22577943 10.1021/cb300151m PMC3560293

[20] Zhang M, Sala C, Hartkoorn RC, Dhar N, Mendoza-Losana A, et al (2012) Streptomycin-starved *Mycobacterium tuberculosis* 18b, a drug discovery tool for latent tuberculosis. Antimicrob Agents Chemother 56: 5782–5789. 22926567 10.1128/AAC.01125-12 PMC3486556

