# Supplementary figures and images for: Improved BM212 MmpL3 Inhibitor Analogue Shows Efficacy in Acute Murine Model of Tuberculosis Infection
Source: PLoS One. 2013 Feb 21;8(2):e56980. doi: 10.1371/journal.pone.0056980 (PMC3578785; doi:10.1371/journal.pone.0056980)

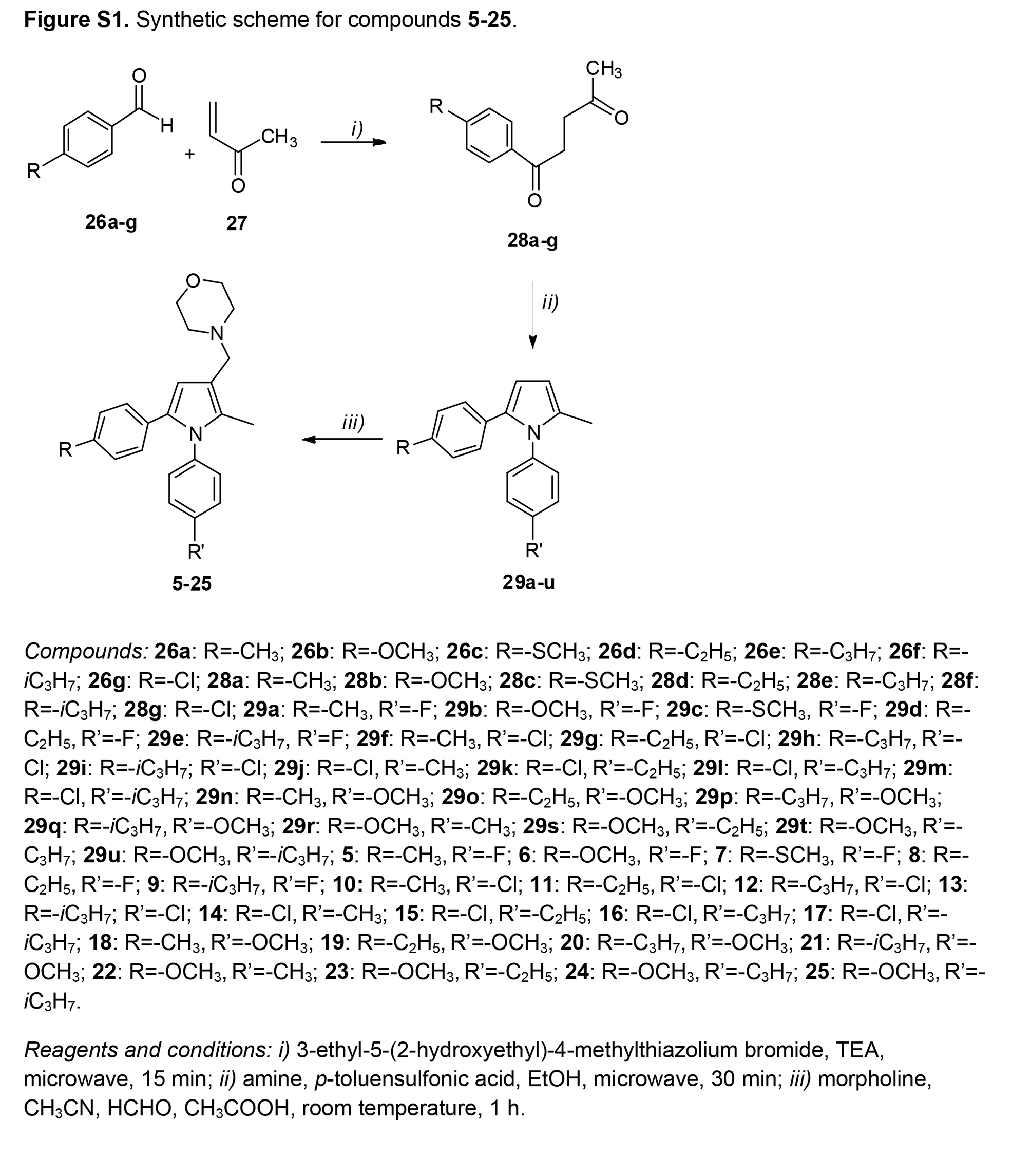

Supplement: Figure S1 — Synthetic scheme for compounds 5–25. (TIF) [file pone.0056980.s001.tif]
