# Supplementary material for: Improved BM212 MmpL3 Inhibitor Analogue Shows Efficacy in Acute Murine Model of Tuberculosis Infection
Source: PLoS One. 2013 Feb 21;8(2):e56980. doi: 10.1371/journal.pone.0056980 (PMC3578785; doi:10.1371/journal.pone.0056980)
Supplement: Table S1 — Oligonucleotides used in this work. (PDF) [file pone.0056980.s002.pdf]

Table S1. Oligonucleotides used in this work.

Name	Sequence	Purpose
MMPLB1	GACGTGTGTGACAACCAAA	Amplification and sequencing of the *M. tuberculosis* H37Rv and *M. bovis* BCG *mmpL3* gene
MMPLB10	AGGTGCTGCTGGGCGGGAA	
MMPLB4	AACACGGTGATAGACAGGAT	Sequencing of the *M. tuberculosis* H37Rv and *M. bovis* BCG *mmpL3* gene
MMPLB5	ATCCTGTCTATCACCGTGTTG	
MMPLB8	CCGAGCAGCTTCATCACCGA	
MMPLB9	TCGGTGATGAAGCTGCTCGG	
**Name**	**Sequence**	**Purpose**
MMPLB1	GACGTGTGTGACAACCAAA	Amplification and sequencing of the *M. tuberculosis* H37Rv and *M. bovis* BCG *mmpL3* gene
MMPLB10	AGGTGCTGCTGGGCGGGAA	
MMPLB4	AACACGGTGATAGACAGGAT	Sequencing of the *M. tuberculosis* H37Rv and *M. bovis* BCG *mmpL3* gene
MMPLB5	ATCCTGTCTATCACCGTGTTG	
MMPLB8	CCGAGCAGCTTCATCACCGA	
MMPLB9	TCGGTGATGAAGCTGCTCGG	
**Name**	**Sequence**	**Purpose**
MMPLB1	GACGTGTGTGACAACCAAA	Amplification and sequencing of the *M. tuberculosis* H37Rv and *M. bovis* BCG *mmpL3* gene
MMPLB10	AGGTGCTGCTGGGCGGGAA	
MMPLB4	AACACGGTGATAGACAGGAT	Sequencing of the *M. tuberculosis* H37Rv and *M.*
MMPLB5	ATCCTGTCTATCACCGTGTTG	

MMPLB8	CCGAGCAGCTTCATCACCGA	*bovis* BCG *mmpL3* gene
MMPLB9	TCGGTGATGAAGCTGCTCGG	
