# Supplementary material for: Improved BM212 MmpL3 Inhibitor Analogue Shows Efficacy in Acute Murine Model of Tuberculosis Infection
Source: PLoS One. 2013 Feb 21;8(2):e56980. doi: 10.1371/journal.pone.0056980 (PMC3578785; doi:10.1371/journal.pone.0056980)
Supplement: Protocol S1 — Procedure for preparation of compounds 5–25. Physicochemical data of compounds 5–25. (PDF) [file pone.0056980.s003.pdf]

Protocol S1. Procedure for preparation of compounds 5-25. Physicochemical data of compounds 5-25.

Chemistry. All chemicals used were of reagent grade. Yields refer to purified products and are not optimized. A CEM Discovery microwave system apparatus was used for the synthesis of compounds **28a-g** and **29a-u** Melting points were determined in open capillaries on a Gallenkamp apparatus and are uncorrected. Sigma-Aldrich silica gel 60 (230−400 mesh) was used for column chromatography. Merck TLC plates (silica gel 60 F254) were used for thin-layer chromatography (TLC). Sigma-Aldrich aluminum oxide (activity II−III, according to Brockmann) was used for chromatographic purifications. Sigma-Aldrich Stratocrom aluminum oxide plates with a fluorescent indicator were used for TLC to check the purity of the compounds. ^{13}C NMR and ^{1}H NMR spectra were recorded with a Bruker AC 400 spectrometer in the indicated solvent (TMS as the internal standard). The values of the chemical shifts are expressed in ppm.

Compounds **5-25** were prepared as shown in Scheme 1. Briefly, by reacting the suitable benzaldehyde **26a-g** with methyl vinyl ketone **27** in the Discovery Microwave System apparatus 1,4-diketones **28a-g** were obtained. Then by cyclization of **28a-g** in the presence of the appropriate amine, the expected 1,5-diarylpyrroles **29a-u** were obtained. Finally, the synthesis of compounds **5-25** were achieved by reacting compounds **29a-u** with formaldehyde and morpholine, following Mannich reaction conditions.

General procedure for the preparation of pentane-1,4-diones 28a-g. Benzaldehyde **26a-g** (0.09 mol), triethylamine (19.5 mL, 0.14mol), methyl vinyl ketone **27** (0.09 mol), and 3-ethyl-5-(2-hydroxyethyl)-4-methylthiazolium bromide (3.53 g, 0.014 mol) were mixed together in a round-bottom flask equipped with a stirring bar. The flask was heated in the cavity of a Discovery Microwave System apparatus (150W for 15 min, internal temperature 70° C, and internal pressure 60 psi). The residue was stirred with 10 ml of 2N HCl for 30 min. After extraction with ethyl acetate, the organic layer was washed with aqueous sodium bicarbonate and water. The organic fractions were dried over Na_2SO_4, filtered, and concentrated to give a crude orange liquid. Chromatography on aluminum oxide (activity II–III, according to Brockmann) (cyclohexane/ethyl acetate, 3:1 v/v) gave the desired **28a-g** were yielded as light-yellow solids which, after recrystallization from cyclohexane, gave an analytical sample as needles.

1-[4-(Methyl)phenyl]-pentane-1,4-dione (28a). White needles (yield 60%). Analytical data, mp, and ^{1}H NMR spectrum were consistent with literature [1].

1-[4-(Methoxy)phenyl]-pentane-1,4-dione (28b). Yellowish needles (yield 75%). Analytical data, mp, and ^{1}H NMR spectrum were consistent with literature [2].

1-[4-(Methylthio)phenyl]-pentane-1,4-dione (28c). Yellowish needles (yield 82%). Analytical data, mp, and ^{1}H NMR spectrum were consistent with literature [3].

1-[4-(Ethyl)phenyl]-pentane-1,4-dione (28d). Yellowish oil (yield 60%). Analytical data, mp, and ^{1}H NMR spectrum were consistent with literature [4].

1-[4-(Propyl)phenyl]-pentane-1,4-dione (28e). Yellowish oil (yield 63%). Analytical data, mp, and 1H NMR spectrum were consistent with literature [4].

1-[4-(*i*-Propyl)phenyl]-pentane-1,4-dione (28f). Yellowish oil (yield 68%). Analytical data, mp, and ^{1}H NMR spectrum were consistent with literature [4].

1-[4-(chloro)phenyl]-pentane-1,4-dione (28g). White needles (yield 78%). Analytical data, mp, and ^{1}H NMR spectrum were consistent with literature [4].

General procedure for the preparation of 1,5-diarylpyrroles 29a-u. The proper diketone **28** (2.28 mmol) and the suitable amine (2.28 mmol) were dissolved in ethanol (2 mL) in a round-bottom flask equipped with a stirring bar in the presence of *p*-toluenesulfonic acid (30 mg, 0.17 mmol). The flask was heated in the cavity of the Discovery Microwave System apparatus (150W for 30 min, internal temperature 160° C, and internal pressure 150 psi). At the end the reaction mixture was cooled down

and concentrated. The crude material was purified by chromatography on aluminum oxide (activity II–III, according to Brockmann) with cyclohexane as the eluant to give the expected 1,5-diarylpyrroles **29a-u** as solids in satisfactory yields.
2-Methyl-5-[4-(methyl)phenyl]-1-[4-(fluoro)phenyl]-1H-pyrrole (29a). White needles (yield 70%). Analytical data, mp, and ^{1}H NMR spectrum were consistent with literature [5].
2-Methyl-5-[4-(methoxy)phenyl]-1-[4-(fluoro)phenyl]-1H-pyrrole (29b). White needles (yield 70%). Analytical data, mp, and 1H NMR spectrum were consistent with literature [6].
2-Methyl-5-[4-(methylthio)phenyl]-1-[4-(fluoro)phenyl]-1H-pyrrole (29c). White needles (yield 93%). Analytical data, mp, and 1H NMR spectrum were consistent with literature [3].
2-Methyl-5-[4-(ethyl)phenyl]-1-[4-(fluoro)phenyl]-1H-pyrrole (29d). White needles (yield 75%). Analytical data, mp, and 1H NMR spectrum were consistent with literature [4].
2-Methyl-5-[4-(*i*-propyl)phenyl]-1-[4-(fluoro)phenyl]-1H-pyrrole (29e).White needles (yield 81%). Analytical data, mp, and 1H NMR spectrum were consistent with literature [4].
2-Methyl-5-[4-(methyl)phenyl]-1-[4-(chloro)phenyl]-1H-pyrrole (29f). White needles (yield 78%). Analytical data, mp, and 1H NMR spectrum were consistent with literature [4].
2-Methyl-5-[4-(ethyl)phenyl]-1-[4-(chloro)phenyl]-1H-pyrrole (29g). White needles (yield 90%). Analytical data, mp, and 1H NMR spectrum were consistent with literature [4].
2-Methyl-5-[4-(propyl)phenyl]-1-[4-(chloro)phenyl]-1H-pyrrole (29h). White needles (yield 65%). Analytical data, mp, and 1H NMR spectrum were consistent with literature [4].
2-Methyl-5-[4-(*i*-propyl)phenyl]-1-[4-(chloro)phenyl]-1H-pyrrole (29i).White needles (yield 74%). Analytical data, mp, and 1H NMR spectrum were consistent with literature [4].
2-Methyl-5-[4-(chloro)phenyl]-1-[4-(methyl)phenyl]-1H-pyrrole (29j). White needles (yield 78%). Analytical data, mp, and 1H NMR spectrum were consistent with literature [4].
2-Methyl-5-[4-(chloro)phenyl]-1-[4-(ethyl)phenyl]-1H-pyrrole (29k). White needles (yield 88%). Analytical data, mp, and 1H NMR spectrum were consistent with literature [4].
2-Methyl-5-[4-(chloro)phenyl]-1-[4-(propyl)phenyl]-1H-pyrrole (29l). White needles (yield 70%). Analytical data, mp, and 1H NMR spectrum were consistent with literature [4].
2-Methyl-5-[4-(chloro)phenyl]-1-[4-(*i*-propyl)phenyl]-1H-pyrrole (29m).White needles (yield 60%). Analytical data, mp, and 1H NMR spectrum were consistent with literature [4].
2-Methyl-5-[4-(methyl)phenyl]-1-[4-(methoxy)phenyl]-1H-pyrrole (29n). White needles (yield 58%). Analytical data, mp, and 1H NMR spectrum were consistent with literature [6].
2-Methyl-5-[4-(ethyl)phenyl]-1-[4-(methoxy)phenyl]-1H-pyrrole (29o). White needles (yield 60%). Analytical data, mp, and 1H NMR spectrum were consistent with literature [6].
2-Methyl-5-[4-(propyl)phenyl]-1-[4-(methoxy)phenyl]-1H-pyrrole (29p). White needles (yield 65%). Analytical data, mp, and 1H NMR spectrum were consistent with literature [6].
2-Methyl-5-[4-(*i*-propyl)phenyl]-1-[4-(methoxy)phenyl]-1H-pyrrole (29q). White needles (yield 55%). Analytical data, mp, and 1H NMR spectrum were consistent with literature [6].
2-Methyl-5-[4-(methoxy)phenyl]-1-[4-(methyl)phenyl]-1H-pyrrole (29r). White needles (yield 70%). Analytical data, mp, and 1H NMR spectrum were consistent with literature [6].
2-Methyl-5-[4-(methoxy)phenyl]-1-[4-(ethyl)phenyl]-1H-pyrrole (29s). White needles (yield 68%). Analytical data, mp, and 1H NMR spectrum were consistent with literature [6].
2-Methyl-5-[4-(methoxy)phenyl]-1-[4-(propyl)phenyl]-1H-pyrrole (29t). White needles (yield 58%). Analytical data, mp, and 1H NMR spectrum were consistent with literature [6].
2-Methyl-5-[4-(methoxy)phenyl]-1-[4-(*i*-propyl)phenyl]-1H-pyrrole (29u). White needles (yield 70%). Analytical data, mp, and 1H NMR spectrum were consistent with literature [6].
General procedure for the preparation of compounds 5-25. To a stirred solution of the appropriate pyrrole **29** (5.6 mmol) in acetonitrile (20 mL), a mixture of morpholine (0.57 g, 5.6 mmol),

formaldehyde (0.18 g, 5.6 mmol) (40% in water), and 5 mL of glacial acetic acid was added drop-wise in 5 min. Following addition, the mixture was stirred at room temperature for 1 h and then treated with a solution of sodium hydroxide (20%, w/v) and extracted with ethyl acetate. The organic extracts were combined, washed with brine, and dried over Na_2SO_4. The residue obtained after solvent evaporation was purified by column chromatography, using silica gel and petroleum ether/ethyl acetate (3:1 v/v) to give **5–25** as solids in satisfactory yields. Recrystallization from diethyl ether gave compounds **5–25** as solids in satisfactory yields.

2-Methyl-3-[(morpholin-4-yl)-methyl]-5-[4-(methyl)phenyl]-1-[4-(fluoro)phenyl]-1H-pyrrole (5). White powder, mp 133−135 °C (yield 60%), ^{1}HNMR (400 MHz, $CDCl_3$) δ (ppm): 7.10 (m, 4H), 6.94 (m, 4H), 6.31 (s, 1H), 3.75 (s broad, 4H), 3.45 (s, 2H), 2.54 (s broad, 4H), 2.25(s, 3H), 2.07 (s, 3H). ^{13}C NMR (100 MHz, $CDCl_3$) δ (ppm): 11.02 (CH_3 pyrrolic), 21.01 (CH_3 phenyl), 53.48 (CH_2-N-CH_2), 55.13 (CH_2 pyrrolic), 67.20 (CH_2-O-CH_2), 110.08, 115.74, 116.5, 127.5, 128.7, 129.55, 129.71, 130.54, 132.43, 133.24, 135.09, 160.6. MS-ESI: m/z 387.18 (M + Na^+). HPLC Analysis (Method A): >98% pure (t_R = 2.50 min).

2-Methyl-3-[(morpholin-4-yl)-methyl]-5-[4-(methoxy)phenyl]-1-[4-(fluoro)phenyl]-1H-pyrrole (6) White powder, mp 169 °C (yield 50%) ^{1}HNMR (400 MHz, $CDCl_3$) δ (ppm): 7.08 (m, 4H), 6.95 (m, 2H), 6.69 (m, 2H), 6.27 (s, 1H), 3.75 (m, 7H), 3.45 (s, 2H), 2.50 (s broad, 4H), 2.06 (s, 3H). ^{13}C NMR (100 MHz, $CDCl_3$) δ (ppm): 11.02 (CH_3 pyrrolic), 53.48 (CH_2-N-CH_2), 55.13 (CH_2 pyrrolic), 55.8 (O-CH_3), 67.20 (CH_2-O-CH_2), 110.08, 114.08, 116.5, 127.5, 128.7, 129.55, 129.71, 130.54, 132.43, 133.24, 160.6, 161.5. MS-ESI: m/z 403.18 (M + Na^+). HPLC Analysis (Method A): >98% pure (t_R = 2.42 min).

2-Methyl-3-[(morpholin-4-yl)-methyl]-5-[4-(methylthio)phenyl]-1-[4-(fluoro)phenyl]-1H-pyrrole (7) White powder, mp 134 °C (yield 55%) ^{1}HNMR (400 MHz, $CDCl_3$) δ (ppm): 7.11-7.02 (m, 6H), 6.94 (m, 2H), 6.36 (s, 1H), 3.82 (s broad, 4H), 3.47 (s, 2H), 2.53 (s broad, 4H), 2.42(s, 3H), 2.08 (s, 3H). ^{13}C NMR (100 MHz, $CDCl_3$) δ (ppm): 11.02 (CH_3 pyrrolic), 53.48 (CH_2-N-CH_2), 55.13 (CH_2 pyrrolic), 67.20 (CH_2-O-CH_2), 110.08, 116.5, 127.5, 128.7, 129.55, 129.71, 130.54, 132.43, 133.24, 135.09, 139.4, 160.6. MS-ESI: m/z 387.18 (M + Na^+). HPLC Analysis (Method A): >98% pure (t_R = 2.58 min).

2-Methyl-3-[(morpholin-4-yl)-methyl]-5-[4-(ethyl)phenyl]-1-[4-(fluoro)phenyl]-1H-pyrrole (8) White powder, mp 107 °C (yield 52%) ^{1}HNMR (400 MHz, $CDCl_3$) δ (ppm): 7.10 (m, 4H), 6.96 (m, 4H), 6.32 (s, 1H), 3.77 (m, 4H), 3.48 (s, 2H), 2.56 (m, 6H), 2.07 (s, 3H), 1.18 (t, 3H). ^{13}C NMR (100 MHz, $CDCl_3$) δ (ppm): 11.02 (CH_3 pyrrolic), 14.5 (CH_2-$\underline{C}H_3$), 28.21 ($\underline{C}H_2$-CH_3), 53.48 (CH_2-N-CH_2), 55.13 (CH_2 pyrrolic), 67.20 (CH_2-O-CH_2), 110.08, 116.5, 127.5, 128.7, 129.55, 129.71, 130.1, 130.54, 132.43, 133.24, 135.09, 160.6. MS-ESI: m/z 401.20 (M + Na^+). HPLC Analysis (Method B): >98% pure (t_R = 1.39 min).

2-Methyl-3-[(morpholin-4-yl)-methyl]-5-[4-(*i*-propyl)phenyl]-1-[4-(fluoro)phenyl]-1H-pyrrole (9) White powder, mp 120 °C (yield 55%) ^{1}HNMR (400 MHz, $CDCl_3$) δ (ppm): 7.11-6.93 (m, 8H), 6.32 (s, 1H), 3.73 (m, 4H), 3.43 (s, 2H), 2.80 (m, 1H), 2.52 (s broad, 4H), 2.05 (s, 3H), 1.18 (d, 6H). ^{13}C NMR (100 MHz, $CDCl_3$) δ (ppm): 11.05 (CH_3 pyrrolic), 23.3 (CH-$\underline{C}H_3$), 33.2 ($\underline{C}$H-CH_3), 53.48 (CH_2-N-CH_2), 55.13 (CH_2 pyrrolic), 67.20 (CH_2-O-CH_2), 110.10, 116.5, 127.5, 128.7, 129.55, 129.71, 130.54, 132.43, 133.24, 135.09, 148.4, 160.6. MS-ESI: m/z 415.27 (M + Na^+). HPLC Analysis (Method A): >98% pure (t_R = 2.66 min).

2-Methyl-3-[(morpholin-4-yl)-methyl]-5-[4-(methyl)phenyl]-1-[4-(chloro)phenyl]-1H-pyrrole (10) White powder, mp 130 °C (yield 60%) ^{1}HNMR (400 MHz, $CDCl_3$) δ (ppm): 7.33 (m, 2H), 7.07 (m, 2H), 6.94 (m, 4H), 6.31 (s, 1H), 3.74 (m, 4H), 3.44 (s, 2H), 2.53 (s broad, 4H), 2.26(d, 3H), 2.07 (s, 3H). ^{13}C NMR (100 MHz, $CDCl_3$) δ (ppm): 11.02 (CH_3 pyrrolic), 21.01 (CH_3 phenyl), 53.48 (CH_2-N-CH_2), 55.13 (CH_2 pyrrolic), 67.20 (CH_2-O-CH_2), 110.08, 127.5, 128.7, 129.4, 129.55, 129.6, 129.71,

130.54, 131.1, 132.43, 133.24, 135.09. MS-ESI: m/z 403.16 (M + Na^+). HPLC Analysis (Method A): >98% pure (t_R = 2.60 min).

2-Methyl-3-[(morpholin-4-yl)-methyl]-5-[4-(ethyl)phenyl]-1-[4-(chloro)phenyl]-1H-pyrrole (11) White powder, mp 134 °C (yield 35%) ^{1}HNMR (400 MHz, $CDCl_3$) δ (ppm): 7.33 (m, 2H), 7.08 (m, 2H), 6.95 (m, 4H), 6.32 (s, 1H), 3.74 (m, 4H), 3.44 (s, 2H), 2.55 (m, 6H), 2.07 (s, 3H), 1.18 (t, 3H). ^{13}C NMR (100 MHz, $CDCl_3$) δ (ppm): 11.08 (CH_3 pyrrolic), 14.6 (CH_2-$\underline{C}H_3$), 28.21 ($\underline{C}H_2$-CH_3), 53.48 (CH_2-N-CH_2), 55.13 (CH_2 pyrrolic), 67.20 (CH_2-O-CH_2), 110.09, 127.5, 128.7, 129.4, 129.55, 129.6, 129.71, 130.54, 132.43, 133.24, 135.09, 148.4. MS-ESI: m/z 417.17 (M + Na^+). HPLC Analysis (Method B): >98% pure (t_R = 1.48 min).

2-Methyl-3-[(morpholin-4-yl)-methyl]-5-[4-(propyl)phenyl]-1-[4-(chloro)phenyl]-1H-pyrrole (12) White powder, mp 113 °C (yield 65%) ^{1}HNMR (400 MHz, $CDCl_3$) δ (ppm): 7.32 (m, 2H), 7.07 (m, 2H), 6.93 (m, 4H), 6.32 (s, 1H), 3.75 (m, 4H), 3.43 (s, 2H), 2.50 (m, 6H), 2.07 (s, 3H), 1.58 (m, 2H), 0.90 (t, 3H). ^{13}C NMR (100 MHz, $CDCl_3$) δ (ppm): 11.02 (CH_3 pyrrolic), 13.8 (CH_2-CH_2-$\underline{C}H_3$), 24.1 (CH_2-$\underline{C}H_2$-CH_3), 37.9 ($\underline{C}H_2$-CH_2-CH_3), 53.48 (CH_2-N-CH_2), 55.13 (CH_2 pyrrolic), 67.20 (CH_2-O-CH_2), 110.08, 127.5, 128.7, 129.4, 129.55, 129.6, 129.71, 130.54, 132.43, 133.24, 135.09, 142.0. MS-ESI: m/z 431.19 (M + Na^+). HPLC Analysis (Method B): >98% pure (t_R = 1.58 min).

2-Methyl-3-[(morpholin-4-yl)-methyl]-5-[4-(*i*-propyl)phenyl]-1-[4-(chloro)phenyl]-1H-pyrrole (13) White powder, mp 135 °C (yield 45%) ^{1}HNMR (400 MHz, $CDCl_3$) δ (ppm): 7.34 (m, 2H), 7.09 (m, 2H), 7.01 (m, 2H), 6.95 (m, 2H), 6.32 (s, 1H), 3.74 (m, 4H), 3.44 (s, 2H), 2.81 (m, 1H), 2.53 (s broad, 4H), 2.07 (s, 3H), 1.19 (d, 6H). ^{13}C NMR (100 MHz, $CDCl_3$) δ (ppm): 11.07 (CH_3 pyrrolic), 23.3 (CH-$\underline{C}H_3$), 33.2 ($\underline{C}$H-CH_3), 53.50 (CH_2-N-CH_2), 55.13 (CH_2 pyrrolic), 67.45 (CH_2-O-CH_2), 110.11, 127.5, 128.7, 129.4, 129.55, 129.6, 129.71, 130.54, 132.43, 133.24, 135.09, 144.33. MS-ESI: m/z 431.19 (M + Na^+). HPLC Analysis (Method B): >98% pure (t_R = 1.54 min).

2-Methyl-3-[(morpholin-4-yl)-methyl]-5-[4-(chloro)phenyl]-1-[4-(methyl)phenyl]-1H-pyrrole (14) White powder, mp 111 °C (yield 78%) ^{1}HNMR (400 MHz, $CDCl_3$) δ (ppm): 7.17 (m, 2H), 7.08 (m, 2H), 7.00 (m, 2H), 6.96 (m, 2H), 6.35 (s, 1H), 3.75 (m, 4H), 3.45 (s, 2H), 2.54 (s broad, 4H), 2.38 (s, 3H), 2.06 (s, 3H). ^{13}C NMR (100 MHz, $CDCl_3$) δ (ppm): 11.02 (CH_3 pyrrolic), 21.05 (CH_3 phenyl), 53.60 (CH_2-N-CH_2), 55.13 (CH_2 pyrrolic), 67.23 (CH_2-O-CH_2), 110.08, 122.7, 127.5, 128.7, 128.91, 129.3, 129.6, 132.43, 133.24, 134.3, 135.09, 135.2. MS-ESI: m/z 403.16 (M + Na^+). HPLC Analysis (Method B): >98% pure (t_R = 1.45 min).

2-Methyl-3-[(morpholin-4-yl)-methyl]-5-[4-(chloro)phenyl]-1-[4-(ethyl)phenyl]-1H-pyrrole (15) White powder, mp 102 °C (yield 50%) ^{1}HNMR (400 MHz, $CDCl_3$) δ (ppm): 7.19 (m, 2H), 7.08 (m, 2H), 7.03 (m, 2H), 6.95 (m, 2H), 6.36 (s, 1H), 3.76 (m, 4H), 3.46 (s, 2H), 2.68 (q, 2H), 2.55 (s broad, 4H), 2.07 (s, 3H), 1.26 (t, 3H). ^{13}C NMR (100 MHz, $CDCl_3$) δ (ppm): 11.10 (CH_3 pyrrolic), 14.4 (CH_2-$\underline{C}H_3$), 28.25 ($\underline{C}H_2$-CH_3), 53.48 (CH_2-N-CH_2), 55.15 (CH_2 pyrrolic), 67.20 (CH_2-O-CH_2), 110.08, 127.5, 128.7, 129.3, 129.4, 129.6, 129.71, 130.54, 132.43, 133.24, 135.09, 142.3. MS-ESI: m/z 417.17 (M + Na^+). HPLC Analysis (Method A): >98% pure (t_R = 2.68 min).

2-Methyl-3-[(morpholin-4-yl)-methyl]-5-[4-(chloro)phenyl]-1-[4-(propyl)phenyl]-1H-pyrrole (16) White powder, mp 94 °C (yield 52%) ^{1}HNMR (400 MHz, $CDCl_3$) δ (ppm): 7.17 (m, 2H), 7.07 (m, 2H), 7.03 (m, 2H), 6.95 (m, 2H), 6.36 (s, 1H), 3.75 (m, 4H), 3.44 (s, 2H), 2.62 (t, 2H), 2.53 (s broad, 4H), 2.08 (s, 3H), 1.67 (q, 2H), 0.95 (t, 3H). ^{13}C NMR (100 MHz, $CDCl_3$) δ (ppm): 11.02 (CH_3 pyrrolic), 13.2 (CH_2-CH_2-$\underline{C}H_3$), 23.9 (CH_2-$\underline{C}H_2$-CH_3), 35.4 ($\underline{C}H_2$-CH_2-CH_3), 53.48 (CH_2-N-CH_2), 55.13 (CH_2 pyrrolic), 67.26 (CH_2-O-CH_2), 110.08, 127.5, 128.5, 129.4, 129.6, 129.7, 129.9, 130.54, 132.43, 133.24, 135.09, 138.8. MS-ESI: m/z 431.19 (M + Na^+). HPLC Analysis (Method A): >95% pure (t_R = 2.77 min).

2-Methyl-3-[(morpholin-4-yl)-methyl]-5-[4-(chloro)phenyl]-1-[4-(*i*-propyl)phenyl]-1H-pyrrole (17) White powder, mp 113 °C (yield 60%) ^{1}HNMR (400 MHz, $CDCl_3$) δ (ppm): 7.07 (m, 2H), 7.04

(m, 4H), 6.94 (m, 2H), 6.35 (s, 1H), 3.74 (m, 4H), 3.44 (s, 2H), 2.94 (m, 1H), 2.53 (s broad, 4H), 2.07 (s, 3H), 1.27 (d, 6H). ^{13}C NMR (100 MHz, $CDCl_3$) δ (ppm): 11.02 (CH_3 pyrrolic), 23.5 (CH-$\underline{C}H_3$), 33.8 ($\underline{C}$H-CH_3), 53.48 (CH_2-N-CH_2), 55.13 (CH_2 pyrrolic), 67.20 (CH_2-O-CH_2), 110.08, 127.4, 128.55, 129.3, 129.55, 129.6, 129.71, 130.54, 132.43, 133.24, 135.09, 145.2. MS-ESI: m/z 431.19 (M + Na^+). HPLC Analysis (Method A): >98% pure (t_R = 2.75 min).

2-Methyl-3-[(morpholin-4-yl)-methyl]-5-[4-(methyl)phenyl]-1-[4-(methoxy)phenyl]-1H-pyrrole (18) White powder, mp 128 °C (yield 67%) ^{1}HNMR (400 MHz, $CDCl_3$) δ (ppm): 7.06 (m, 2H), 6.94 (m, 4H), 6.87 (m, 2H), 6.31 (s, 1H), 3.82 (s, 3H), 3.75 (m, 4H), 3.46 (s, 2H), 2.54 (s broad, 4H), 2.24 (d, 3H), 2.05 (s, 3H). ^{13}C NMR (100 MHz, $CDCl_3$) δ (ppm): 11.02 (CH_3 pyrrolic), 21.01 (CH_3 phenyl), 53.48 (CH_2-N-CH_2), 55.13 (CH_2 pyrrolic), 67,20 (CH_2-O-CH_2), 110.08, 114.9, 127.5, 128.7, 128.91, 129.3, 129.6, 132.43, 133.24, 134.3, 142.09, 157.4. MS-ESI: m/z 399.20 (M + Na^+). HPLC Analysis (Method B): >98% pure (t_R = 1.30 min).

2-Methyl-3-[(morpholin-4-yl)-methyl]-5-[4-(ethyl)phenyl]-1-[4-(methoxy)phenyl]-1H-pyrrole (19) White powder, mp 101 °C (yield 50%) ^{1}HNMR (400 MHz, $CDCl_3$) δ (ppm): 7.08 (m, 2H), 6.99 (m, 4H), 6.88 (m, 2H), 6.32 (s, 1H), 3.83 (s, 3H), 3.74 (m, 4H), 3.44 (s, 2H), 2.55 (m, 6H), 2.05 (s, 3H), 1.17 (t, 3H). ^{13}C NMR (100 MHz, $CDCl_3$) δ (ppm): 11.07 (CH_3 pyrrolic), 14.8 (CH_2-$\underline{C}H_3$), 28.21 ($\underline{C}H_2$-CH_3), 53.55 (CH_2-N-CH_2), 55.13 (CH_2 pyrrolic), 67,26 (CH_2-O-CH_2), 110.08, 114.9, 127.25, 128.68, 128.91, 129.3, 129.6, 132.43, 133.24, 134.3, 142.2, 157.3. MS-ESI: m/z 413.22 (M + Na^+). HPLC Analysis (Method B): >98% pure (t_R = 1.36 min).

2-Methyl-3-[(morpholin-4-yl)-methyl]-5-[4-(propyl)phenyl]-1-[4-(methoxy)phenyl]-1H-pyrrole (20) White powder, mp 107 °C (yield 56%) ^{1}HNMR (400 MHz, $CDCl_3$) δ (ppm): 7.07 (m, 2H), 6.95 (m, 4H), 6.87 (m, 2H), 6.31 (s, 1H), 3.82 (s, 3H), 3.74 (m, 4H), 3.44 (s, 2H), 2.49 (m, 6H), 2.05 (s, 3H), 1.57 (m, 2H), 0.89 (t, 3H). ^{13}C NMR (100 MHz, $CDCl_3$) δ (ppm): 11.07 (CH_3 pyrrolic), 13.5 (CH_2-CH_2-$\underline{C}H_3$), 24.8 (CH_2-$\underline{C}H_2$-CH_3), 37.9 ($\underline{C}H_2$-CH_2-CH_3), 53.48 (CH_2-N-CH_2), 55.13 (CH_2 pyrrolic), 67,20 (CH_2-O-CH_2), 110.09, 114.9, 127.3, 128.68, 128.91, 129.89, 132.43, 133.24, 134.3, 142.2, 143.33, 157.3. MS-ESI: m/z 427.24 (M + Na^+). HPLC Analysis (Method B): >98% pure (t_R = 1.43 min).

2-Methyl-3-[(morpholin-4-yl)-methyl]-5-[4-(*i*-propyl)phenyl]-1-[4-(methoxy)phenyl]-1H-pyrrole (21) White powder, mp 109 °C (yield 52%) ^{1}HNMR (400 MHz, $CDCl_3$) δ (ppm): 7.07 (m, 2H), 6.98 (m, 4H), 6.88 (m, 2H), 6.32 (s, 1H), 3.84 (s, 3H), 3.75 (m, 4H), 3.45 (s, 2H), 2.80 (m, 1H), 2.54 (s broad, 4H), 2.05 (s, 3H), 1.18 (d, 6H). ^{13}C NMR (100 MHz, $CDCl_3$) δ (ppm): 11.09 (CH_3 pyrrolic), 23.44 (CH-$\underline{C}H_3$), 33.78 ($\underline{C}$H-CH_3), 53.48 (CH_2-N-CH_2), 55.13 (CH_2 pyrrolic), 67,26 (CH_2-O-CH_2), 110.08, 114.9, 127.3, 128.29, 128.91, 129.9, 132.43, 133.34, 134.3, 142.35, 148.4, 157.3. MS-ESI: m/z 427.24 (M + Na^+). HPLC Analysis (Method B): >98% pure (t_R = 1.40 min).

2-Methyl-3-[(morpholin-4-yl)-methyl]-5-[4-(methoxy)phenyl]-1-[4-(methyl)phenyl]-1H-pyrrole (22) White powder, mp 115 °C (yield 76%) ^{1}HNMR (400 MHz, $CDCl_3$) δ (ppm): 7.15 (d, 2H), 6.98 (m, 4H), 6.67 (m, 2H), 6.26 (s, 1H), 3.75 (m, 7H), 3.46 (s, 2H), 2.58 (s broad, 4H), 2.39 (d, 3H), 2.06 (s, 3H). ^{13}C NMR (100 MHz, $CDCl_3$) δ (ppm): 11.02 (CH_3 pyrrolic), 21.01 (CH_3 phenyl), 53.48 (CH_2-N-CH_2), 55.13 (CH_2 pyrrolic), 67,20 (CH_2-O-CH_2), 110.10, 114.9, 127.6, 128.7, 128.91, 129.3, 129.6, 132.43, 133.24, 135.2, 148, 160.6. MS-ESI: m/z 399.20 (M + Na^+). HPLC Analysis (Method B): >98% pure (t_R = 1.30 min).

2-Methyl-3-[(morpholin-4-yl)-methyl]-5-[4-(methoxy)phenyl]-1-[4-(ethyl)phenyl]-1H-pyrrole (23) White powder, mp 72 °C (yield 52%) ^{1}HNMR (400 MHz, $CDCl_3$) δ (ppm): 7.17 (d, 2H), 7.04 (d, 2H), 6.97 (d, 2H), 6.67 (d, 2H), 6.27 (s, 1H), 3.75 (m, 7H), 3.45 (s, 2H), 2.68 (q, 2H), 2.54 (s broad, 4H), 2.07 (s, 3H), 1.26 (t, 3H). ^{13}C NMR (100 MHz, $CDCl_3$) δ (ppm): 11.02 (CH_3 pyrrolic), 14.6 (CH_2-$\underline{C}H_3$), 28.33 ($\underline{C}H_2$-CH_3), 53.48 (CH_2-N-CH_2), 55.13 (CH_2 pyrrolic), 67,27 (CH_2-O-CH_2), 110.08,

114.9, 127.3, 128.7, 128.91, 129.3, 129.6, 132.43, 133.24, 141.1, 147.8, 161. MS-ESI: m/z 413.22 (M + Na^+). HPLC Analysis (Method A): >98% pure (t_R = 2.57 min).

2-Methyl-3-[(morpholin-4-yl)-methyl]-5-[4-(methoxy)phenyl]-1-[4-(propyl)phenyl]-1H-pyrrole (24) White powder, mp 90 °C (yield 66%) ^{1}HNMR (400 MHz, $CDCl_3$) δ (ppm): 7.14 (m, 2H), 7.03 (m, 2H), 6.96 (m, 2H), 6.66 (m, 2H), 6.27 (s, 1H), 3.74 (m, 7H), 3.45 (s, 2H), 2.61 (t, 2H), 2.54 (s broad, 4H), 2.07 (s, 3H), 1.66 (m, 2H), 0.93 (t, 3H). ^{13}C NMR (100 MHz, $CDCl_3$) δ (ppm): 11.02 (CH_3 pyrrolic), 13.5 (CH_2-CH_2-$\underline{C}H_3$), 24.2 (CH_2-$\underline{C}H_2$-CH_3), 37.9 ($\underline{C}H_2$-CH_2-CH_3), 53.48 (CH_2-N-CH_2), 55.13 (CH_2 pyrrolic), 67,20 (CH_2-O-CH_2), 110.09, 125.4, 127.4, 128.7, 128.91, 129.9, 132.43, 133.3, 134.3, 138.8, 143.33, 160.8. MS-ESI: m/z 427.24 (M + Na^+). HPLC Analysis (Method A): >98% pure (t_R = 2.64 min).

2-Methyl-3-[(morpholin-4-yl)-methyl]-5-[4-(methoxy)phenyl]-1-[4-(*i*-propyl)phenyl]-1H-pyrrole (25) White powder, mp 88 °C (yield 70%) ^{1}HNMR (400 MHz, $CDCl_3$) δ (ppm): 7.19 (d, 2H), 7.04 (d, 2H), 6.96 (d, 2H), 6.66 (d, 2H), 6.27 (s, 1H), 3.74 (m, 7H), 3.45 (s, 2H), 2.93 (m, 1H), 2.54 (s broad, 4H), 2.07 (s, 3H), 1.26 (t, 3H). ^{13}C NMR (100 MHz, $CDCl_3$) δ (ppm): 11.02 (CH_3 pyrrolic), 23.42 (CH-$\underline{C}H_3$), 33.78 ($\underline{C}$H-CH_3), 53.48 (CH_2-N-CH_2), 55.13 (CH_2 pyrrolic), 67,20 (CH_2-O-CH_2), 110.08, 126.7, 127.3, 128.29, 128.91, 129.9, 132.43, 133.34, 134.3, 145.35, 148.4, 160.7. MS-ESI: m/z 427.24 (M + Na^+). HPLC Analysis (Method B): >98% pure (t_R = 1.41 min).

References.

1. Shridhar DR, Jogibhukta M, Rao P, Shanthan H, Vijay K. (1982) An improved method for the preparation of 2,5-disubstituted thiophenes. Synthesis. 12**:** 1061-1062.
2. Xue S, Li L.-Z, Liu Y.-K, Guo Q.-X. (2006) Zinc-mediated chain extension reaction of 1,3-diketones to 1,4-diketones and diastereoselective synthesis of trans-1,2-disubstituted cyclopropanols. J. Org. Chem. 71: 215-218.
3. Biava M, Porretta GC, Pompei R, Botta M, Manetti F, De Logu A. (2009) Pyrrole compounds as inibitors of mycobacteria, synthesis thereof and intermediates thereto. PCT Int. Appl. 2009, p 31, WO 2009040755 A2 20090402 AN 2009:386048.
4. Biava M, Porretta GC, Poce G, De Logu A, Saddi M, Meleddu R, Manetti F, De Rossi E, Botta M. (2008) 1,5-Diphenyl pyrrole derivatives as antimycobacterial agents. Probing the influence on antimycobacterial activity of lipophylic substituents at the phenyl rings. J. Med. Chem. 51: 3644-3648.
5. Biava M, Porretta GC, Poce G, Supino S, Deidda D, Pompei R, Molicotti P, Manetti F, Botta M. (2006) Antimycobacterial agents. Novel diarylpyrrole derivatives of BM212 endowed with high activity toward *Mycobacterium tuberculosis* and low cytotoxicity. J. Med. Chem. 49: 4946-4952.
6. Biava M, Porretta GC, Poce G, Battilocchio C, Alfonso S, De Logu A, Serra N, Manetti F, Botta M. (2010) Identification of a novel pyrrole derivative endowed with antimycobacterial activity and protection index comparable to that of the current antitubercular drugs streptomycin and rifampin. Bioorg. Med. Chem. 18: 8076-8084.
