# Supplementary material for: Improved BM212 MmpL3 Inhibitor Analogue Shows Efficacy in Acute Murine Model of Tuberculosis Infection
Source: PLoS One. 2013 Feb 21;8(2):e56980. doi: 10.1371/journal.pone.0056980 (PMC3578785; doi:10.1371/journal.pone.0056980)
Supplement: Protocol S2 — HPLC analysis of compounds 5–25. (PDF) [file pone.0056980.s004.pdf]

Protocol S2. HPLC analysis of compounds 5-25.

HPLC-MS analyses were conducted on an Agilent1100 at 40 ºC coupled with a Waters-ZMD2000 mass spectrometer. The method of ionization was alternate-scan Positive and Negative Electrospray. Method A: Sunfire C18 column (30 mm x 2.1 mm i.d. 3.5 µm packing diameter), flow 1.0 mL/min, eluents H_2O-formic acid 0.1% / acetonitrile, ramp 0-0.1 min 100/0, 0.1-3.0 min 100/0 to 10/90, 3.0-3.5 min 10/90, 3.51-4.5 min 100/0. Method B: Acquity UPLC BEH C18 column (50 mm x 3 mm i.d. 1.7 µm packing diameter), flow 0.8 mL/min, eluents H_2O-ammonium acetate 25 mM + 10% acetonitrile at pH 6.6 / acetonitrile, ramp 0-0.2 min 100/0, 0.2-1.0 min 100/0 to 10/90, 1.0-1.8 min 10/90, 1.8-2.0 min 100/0.
