# Supplementary material for: Improved BM212 MmpL3 Inhibitor Analogue Shows Efficacy in Acute Murine Model of Tuberculosis Infection
Source: PLoS One. 2013 Feb 21;8(2):e56980. doi: 10.1371/journal.pone.0056980 (PMC3578785; doi:10.1371/journal.pone.0056980)
Supplement: Protocol S3 — Determination of compounds Chromatographic Hydrophobicity Index (CHI) at acidic, neutral and alkaline pHs. (PDF) [file pone.0056980.s005.pdf]

Protocol S3. Determination of compounds Chromatographic Hydrophobicity Index (CHI) at acidic, neutral and alkaline pHs.

The determination of compounds Chromatographic Hydrophobicity Index (CHI) at acidic, neutral and alkaline pHs was accomplished by retention time measurements using Luna C18 columns obtained from Phenomenex**.** The column dimensions were 50 x 3 mm, particle size, 5 um. Compounds analysed were dissolved in Acetonitrile (0.5 mM) from a stock solution 10 mM in DMSO. Mobile Phase A:50 mM Ammonium Acetate aqueous solution, pH adjusted to 7.4 and 10.5 by addition of concentrated ammonia solution and 0.01M concentrated phosphoric acid in water, pH 2. Mobile Phase B: Acetonitrile. *HPLC Method:* flow rate: 1.0 ml/min, temperature: 30ºC, gradient: 0 to 3.0 min 0 to 100% B solvent***;*** 3.0 to 3.5 min. 100% B solvent (Acetonitrile); 3.5 to 3.7 min. from 100% B to 0% B. Total run time 5 min. *Calibration set of compounds:* Theophylline, Phenyltetrazole, Benzimidazole, Colchicine, Phenyltheophylline, Acetophenone, Indole, Propiophenone, Butyrophenone, Valerophenone. The calculation of the Chromatographic Hydrophobicity data expressed as CHILogD was carried out as previously described [1].

References.

1. Valko K. (2002) Measurement and predictions of physicochemical properties. Eaton Publishing, Westborough, MA.
