# Supplementary material for: Improved BM212 MmpL3 Inhibitor Analogue Shows Efficacy in Acute Murine Model of Tuberculosis Infection
Source: PLoS One. 2013 Feb 21;8(2):e56980. doi: 10.1371/journal.pone.0056980 (PMC3578785; doi:10.1371/journal.pone.0056980)
Supplement: Protocol S4 — Determination of compounds binding to human serum albumin (HSA). (PDF) [file pone.0056980.s006.pdf]

Protocol S4. Determination of compounds binding to human serum albumin (HSA).

The determination of compounds binding to human serum albumin (HSA) was carried out by retention time measurements using immobilized HSA HPLC columns obtained from Chiral Technologies Ltd, France. The column dimensions were 50x3 mm. The mobile phase was 50 mM ammonium acetate buffer pH 7.4 and HPLC grade 2-propanol. *HPLC Method*: flow rate 1.8 ml/min applying 2.5 min 2-propanol gradient up to 30%. From 2.5 min to 4.5 min the 2-propanol concentration in the mobile phase was kept at 30%. From 4.5 min to 4.6 min, the 2-propanol concentration was decreased to 0% and kept like that until the end of the gradient run which was 6 min. A calibration set of compounds was first analyzed for which plasma protein binding data were available: Warfarin, Nizatidine, Bromazepam, Carbamazepine, Budesonide, Nicardipine, Indomethacine, Piroxicam, Naproxen. The calculation of the HSA binding data expressed as %HSA was carried out as described previously [1].

References.

1. Valko K, Nunhuck S, Bevan C, Abraham MH, Reynolds DP. (2003) Fast gradient HPLC method to determine compounds binding to human serum albumin. Relationships with octanol/water and immobilized artificial membrane lipophilicity. Pharm Sci 92: 2236-2248.
