# Supplementary material for: Improved BM212 MmpL3 Inhibitor Analogue Shows Efficacy in Acute Murine Model of Tuberculosis Infection
Source: PLoS One. 2013 Feb 21;8(2):e56980. doi: 10.1371/journal.pone.0056980 (PMC3578785; doi:10.1371/journal.pone.0056980)
Supplement: Protocol S5 — Determination of compounds binding to plasma proteins (PPB). (PDF) [file pone.0056980.s007.pdf]

Protocol S5. Determination of compounds binding to plasma proteins (PPB).

Test compounds in DMSO (200 µL) were mixed with phosphate buffer saline (350 µL) and plasma (250 µL) and dialyzed against PBS (pH 7.4) for 4-6 hours at 37ºC. After incubation, the contents of each plasma and buffer compartment were transferred to a 96-well plate with equal volumes of control buffer or plasma as appropriate to maintain matrix equivalence for analysis. Plasma proteins were then precipitated by the addition of acetonitrile/methanol (80:20) containing internal standard. The plates were centrifuged for 15 min at 3700 rpm (4ºC), and the supernatant was removed for analysis by mass spectrometry (LC-MS/MS) to determine the percentage of test compound in the buffer and plasma chambers from peak areas relative to the internal standard. The percentage bound and unbound was calculated as follows:

%unbound = ([Buffer chamber] / [Plasma chamber) x 100

%PPB = 100 - %Unbound
