# Supplementary material for: Improved BM212 MmpL3 Inhibitor Analogue Shows Efficacy in Acute Murine Model of Tuberculosis Infection
Source: PLoS One. 2013 Feb 21;8(2):e56980. doi: 10.1371/journal.pone.0056980 (PMC3578785; doi:10.1371/journal.pone.0056980)
Supplement: Protocol S6 — Microsomal fraction stability experimental procedure. (PDF) [file pone.0056980.s008.pdf]

Protocol S6. Microsomal fraction stability experimental procedure.

Pooled mouse and human liver microsomes are purchased from Xenotech. Microsomes (final protein concentration 0.5 mg/mL, $MgCl_2$ (final concentration = 5 mM) and test compound (final substrate concentration = 0.5 µM; final DMSO concentration = 0.5%) in 0.1M phosphate buffer pH 7.4 were pre-incubated at 37°C prior to the addition of NADPH (final concentration = 1 mM) to initiate the reaction. The final incubation volume was 600 µL. A control incubation was included for each compound tested where 0.1 M phosphate buffer pH 7.4 is added instead of NADPH (minus NADPH). One control compound was included with each species. All incubations were performed singularly for each test compound. Each compound was incubated for 30 minutes and samples (90 µL) of incubate were taken at 0, 5, 15, 20 and 30 min. The control (minus NADPH) was sampled at 30 min only. The reactions were stopped by the addition of sample to 200 µL Acetonitrile:Methanol 3:1 containing internal standard. The terminated samples were centrifuged at 2.500 rpm for 20 min at 4°C to precipitate the protein.

Quantitative Analysis: following protein precipitation, the samples were analyzed using specific LC-MS/MS conditions.

Data Analysis: from a plot of ln peak area ratio (compound peak area/internal standard peak area) against time, the gradient of the line was determined. Subsequently, half-life and intrinsic clearance were calculated using the equations below:

Elimination rate constant (k) = (- gradient)

Half life ($t_{1/2}$) (min) = $\frac{0.693}{k}$

Intrinsic Clearance (CL_{int}) (mL/min/g protein) = $\frac{V \times 0.693}{t_{1/2}}$

where V=Incubation volume mL/g microsomal protein.
